# Prevalence and Angiographic Severity of Obstructive Coronary Artery Disease in Patients Presenting with a Dilated Cardiomyopathy Phenotype: A Retrospective Single-Center Cohort Study

**DOI:** 10.3390/medicina62081608

**Published:** 2026-08-21

**Authors:** Stefan Yambolov, Rozen Grigorov, Nikolay Nikolov, Ivaylo Borisov, Svetoslav Georgiev

**Affiliations:** 1 Interventional Cardiology Clinic, St. Marina University Hospital, 9010 Varna, Bulgaria; 2 Department of Cardiology and Rheumatology, Medical University—Varna, 9002 Varna, Bulgaria; 3 Faculty of Medicine, Medical University—Varna, 9002 Varna, Bulgaria

**Keywords:** dilated cardiomyopathy, coronary angiography, obstructive coronary artery disease, ischemic cardiomyopathy, heart failure, chronic total occlusion

## Abstract

*Background and Objectives*: Identification of obstructive coronary artery disease (CAD) in patients presenting with a dilated cardiomyopathy phenotype is clinically important because it may influence etiologic classification, prognosis, and subsequent management. However, real-world angiography-based data on the prevalence and angiographic severity of obstructive CAD in this setting remain limited, particularly in regional single-center cohorts. *Materials and Methods:* This retrospective single-center cohort study included consecutive patients presenting with a newly diagnosed dilated cardiomyopathy (DCM) phenotype of initially unknown etiology who underwent first-time diagnostic invasive coronary angiography at St. Marina University Hospital, Varna, Bulgaria, between January 2010 and December 2025. Obstructive CAD was defined as stenosis >70% in a major epicardial coronary artery with a reference vessel diameter >2.5 mm. Patients were categorized as having non-obstructive or no significant coronary stenoses, single-vessel disease, two-vessel disease, or three-vessel disease. Additional descriptive analysis assessed severe occlusive coronary lesions, defined as chronic total occlusions or subtotal stenoses. The primary endpoint was the prevalence of obstructive CAD. Secondary analyses included angiographic disease extent, vessel-level coronary involvement, severe occlusive lesions, and coronary revascularization. *Results:* A total of 107 patients were included. Mean age was 55.4 ± 10.9 years, and 97 patients (90.7%) were male. Obstructive CAD was identified in 19 patients (17.8%; 95% confidence interval [CI], 11.7–26.1%), whereas 88 patients (82.2%) had non-obstructive or no significant coronary stenoses. Among patients with obstructive CAD, 3 (15.8%) had single-vessel disease, 4 (21.1%) had two-vessel disease, and 12 (63.2%) had three-vessel disease. Severe occlusive coronary lesions were present in 10 of 19 patients (52.6%); 8 (42.1%) had at least one chronic total occlusion and 5 (26.3%) had at least one subtotal stenosis. *Conclusions:* In this retrospective single-center cohort of patients presenting with a dilated cardiomyopathy phenotype and referred for coronary angiography, obstructive CAD was present in fewer than one in five patients. In contrast, most had non-obstructive or no significant epicardial coronary stenoses. However, when obstructive CAD was present, three-vessel disease and severe occlusive lesions were frequent. These findings indicate that obstructive epicardial CAD was uncommon in this selected angiography-referred cohort while underscoring the clinical role of coronary angiography when an ischemic or mixed etiology is clinically suspected.

## 1. Introduction

Dilated cardiomyopathy (DCM) is a myocardial disease characterized by left ventricular or biventricular dilatation and systolic dysfunction that cannot be explained solely by abnormal loading conditions, such as hypertension or valvular disease, or by coronary artery disease sufficient to account for the observed phenotype [1,2]. In clinical practice, however, patients may initially present with a DCM phenotype before an ischemic or mixed etiology has been excluded. Identification of obstructive coronary artery disease (CAD) in patients with a DCM phenotype is clinically relevant because it has direct implications for etiologic classification, prognosis, and therapeutic management [3,4]. For this reason, evaluation for possible ischemic heart disease remains an integral part of the diagnostic work-up in patients presenting with DCM and heart failure [1,3,5]. Coronary angiography has traditionally remained the reference method for confirming or excluding obstructive epicardial CAD [3,5].

Real-world data on the burden of angiographically significant CAD among patients with DCM are valuable, particularly in local and regional populations, where referral patterns, thresholds for invasive evaluation, and cardiovascular risk profiles may differ. Published cohort studies have shown that the prevalence of significant CAD in patients with a DCM phenotype varies substantially across populations, supporting the need for center-specific and regional data [6,7]. Such data may help define the expected diagnostic yield of invasive coronary angiography in this setting.

The present study aimed to assess the prevalence and angiographic severity of obstructive coronary artery disease in patients presenting with a dilated cardiomyopathy phenotype and referred for first-time diagnostic coronary angiography in a Bulgarian tertiary-care single-center cohort.

## 2. Materials and Methods

### 2.1. Study Design and Setting

This retrospective, single-center cohort study was conducted at the Interventional Cardiology Clinic of St. Marina University Hospital, Varna, Bulgaria. The study was based on data from the institutional catheterization laboratory database, angiographic documentation, procedural reports, and available clinical records.

### 2.2. Study Population

The study included all consecutive patients presenting with a newly diagnosed dilated cardiomyopathy phenotype of initially unknown etiology who underwent first-time diagnostic invasive coronary angiography between January 2010 and December 2025.

For the purpose of this study, a dilated cardiomyopathy phenotype was operationally defined as left ventricular systolic dysfunction with an LVEF < 50%, together with left ventricular dilatation defined by an LVEDD > 58 mm in men or >52 mm in women, in the absence of abnormal loading conditions sufficient to explain the observed phenotype at the time of initial clinical assessment.

Patients were included if they had newly diagnosed left ventricular dilatation and systolic dysfunction and were referred for invasive coronary angiography to exclude an ischemic or mixed etiology. Patients were excluded if they had a prior coronary angiography assessment or if angiographic documentation was insufficient for classification of coronary artery disease extent.

Referral for invasive coronary angiography was based on individualized clinical assessment by the treating cardiology team, taking into account the overall clinical presentation, cardiovascular risk profile, available non-invasive findings, and patient preference. A single unchanged institutional referral algorithm was not applied throughout the entire study period. In all patients selected for anatomical assessment of the coronary arteries within this cohort, invasive coronary angiography was used rather than coronary computed tomography angiography.

Because the study was derived from the catheterization laboratory database, patients with a newly diagnosed dilated cardiomyopathy phenotype who were not referred for invasive coronary angiography were not included. Therefore, the study population represents a selected angiography-referred cohort and not the entire population of patients with a newly diagnosed dilated cardiomyopathy phenotype evaluated at our institution.

### 2.3. Echocardiographic Assessment

Baseline left ventricular dimensions and systolic function were assessed by transthoracic echocardiography. Left ventricular end-diastolic diameter (LVEDD) was measured using two-dimensional echocardiography predominantly from parasternal long-axis view. Sex-specific LVEDD thresholds (>58 mm in men and >52 mm in women) were used to define left ventricular dilatation. Left ventricular ejection fraction (LVEF) was quantified using the Simpson biplane method in apical four- and two-chamber views. Echocardiographic measurements were obtained as part of the baseline diagnostic evaluation.

### 2.4. Coronary Angiography and Definitions

Coronary angiography was performed according to standard clinical practice. Angiographic findings were obtained from the catheterization laboratory database and verified against procedural reports and available angiographic documentation. Coronary stenosis severity was assessed by visual angiographic estimation supplemented by quantitative coronary angiography (QCA). Intracoronary physiological assessment, including fractional flow reserve (FFR) or instantaneous wave-free ratio (iFR), was not performed.

Patients were classified according to the angiographic extent of coronary artery disease as having non-obstructive or no significant coronary stenoses, single-vessel disease, two-vessel disease, or three-vessel disease. Obstructive coronary artery disease was defined as stenosis >70% in a major epicardial coronary artery with a reference vessel diameter >2.5 mm. Patients categorized as having single-vessel, two-vessel, or three-vessel disease were considered to have obstructive CAD. Patients categorized as having non-obstructive or no significant coronary stenoses were considered not to have obstructive CAD. For descriptive purposes, patients without obstructive CAD were further classified as having angiographically visible non-obstructive coronary atherosclerosis or no angiographic stenoses. Non-obstructive coronary atherosclerosis was defined by luminal irregularities or coronary stenoses not meeting the study definition of obstructive CAD.

For additional descriptive analysis, chronic total occlusions and subtotal stenoses were grouped as severe occlusive coronary lesions. For total coronary occlusions, lesion chronicity was determined by the operator at the time of the index angiography and recorded in the procedural report. The assessment was based on the overall clinical and angiographic picture, including the clinical presentation, ECG findings, lesion morphology, visible thrombus, and the presence of collateral circulation. We used the classification documented in the original report and did not retrospectively reassess the angiographic images. Subtotal stenosis was defined as a critical near-occlusive lesion with preserved antegrade flow, as documented in the angiographic report.

Revascularization strategy following coronary angiography was obtained from the procedural and available clinical documentation and was categorized as PCI, CABG, or medical treatment without revascularization. PCI, when performed, was undertaken during the index angiographic procedure. The exact timing of subsequent CABG was not systematically available in the retrospective documentation and was therefore not analyzed.

### 2.5. Study Objectives

The primary endpoint was the prevalence of obstructive CAD among patients presenting with a dilated cardiomyopathy phenotype and referred for coronary angiography. Secondary analyses included angiographic disease extent, vessel-level coronary involvement, severe occlusive coronary lesions, and coronary revascularization.

### 2.6. Statistical Analysis

Continuous variables are presented as mean ± standard deviation (SD), and categorical variables as absolute numbers and percentages. The distribution of continuous variables was assessed using the Shapiro–Wilk test. Between-group comparisons of approximately normally distributed continuous variables were performed using Welch’s independent-samples *t*-test, whereas the Mann–Whitney U test was used for LVEDD, which differed from normality. Categorical variables were compared using Fisher’s exact test because of the relatively small size of the obstructive CAD subgroup. No imputation was performed; complete data on the baseline variables included in Table 1 were available for all patients. A two-sided *p*-value < 0.05 was considered statistically significant. The prevalence of obstructive CAD was reported with a 95% confidence interval calculated using the Wilson score method. No a priori sample size calculation was performed, as this retrospective study included all eligible consecutive patients during the predefined study period. Statistical analyses were performed using Python version 3.13.5 with NumPy version 2.3.5, pandas version 2.2.3, SciPy version 1.17.0, and statsmodels version 0.14.6.

### 2.7. Ethical Considerations

The study was approved by the Research Ethics Committee of the Medical University “Prof. Dr. Paraskev Stoyanov”—Varna (Decision No. 32/28 May 2026) and was conducted in accordance with the Declaration of Helsinki. The requirement for written informed consent was waived due to the retrospective observational design and the use of anonymized data.

## 3. Results

### 3.1. Baseline Characteristics

A total of 107 consecutive patients presenting with a newly diagnosed dilated cardiomyopathy phenotype who underwent first-time diagnostic invasive coronary angiography between January 2010 and December 2025 were included in the analysis. The mean age of the cohort was 55.4 ± 10.9 years, and 97 patients (90.7%) were male. Hypertension was present in 105 patients (98.1%), dyslipidemia in 81 (75.7%), diabetes mellitus in 22 (20.6%), active smoking in 18 (16.8%), and alcohol use in 19 (17.8%). The mean estimated glomerular filtration rate (eGFR, CKD-EPI) was 82.4 ± 22.5 mL/min/1.73 m^2^, and the mean low-density lipoprotein (LDL) cholesterol was 2.69 ± 1.05 mmol/L. Echocardiographic measurements showed a mean left ventricular end-diastolic diameter of 65 ± 6.4 mm and a mean left ventricular ejection fraction of 33.6 ± 8.4%.

Patients with obstructive CAD had a higher prevalence of diabetes mellitus (42.1% vs. 15.9%, *p* = 0.024) and dyslipidemia (94.7% vs. 71.6%, *p* = 0.039) compared with patients without obstructive CAD. Baseline characteristics are summarized in Table 1.

### 3.2. Angiographic Prevalence of Obstructive Coronary Artery Disease

Among the 107 patients included in the study, 19 patients had obstructive coronary artery disease, corresponding to a prevalence of 17.8% (95% confidence interval [CI], 11.7–26.1%). The remaining 88 patients (82.2%) did not have obstructive CAD. Of these, 27 patients had angiographically visible non-obstructive coronary atherosclerosis, corresponding to 25.2% of the total cohort, whereas 61 patients (57.0%) had no angiographic stenoses. Thus, the majority of patients presenting with a dilated cardiomyopathy phenotype and referred for first-time diagnostic coronary angiography did not have angiographically significant obstructive epicardial CAD. Angiographic distribution of coronary artery disease in the study cohort is summarized in Table 2.

### 3.3. Extent of Vessel Disease

With respect to the angiographic extent of disease, single-vessel disease was present in 3 patients (2.8%), two-vessel disease in 4 patients (3.7%), and three-vessel disease in 12 patients (11.2%). Three-vessel disease was therefore the most frequent pattern among patients with obstructive CAD.

When analyzed within the subgroup of patients with obstructive CAD, 3 of 19 patients (15.8%) had single-vessel disease, 4 of 19 (21.1%) had two-vessel disease, and 12 of 19 (63.2%) had three-vessel disease.

Among the 19 patients with obstructive CAD, the left anterior descending artery was involved in 17 patients (89.5%), the left circumflex artery in 16 (84.2%), and the right coronary artery in 16 (84.2%). Obstructive left main coronary artery disease was present in 1 patient (5.3%).

### 3.4. Severe Occlusive Lesions Within the Obstructive CAD Subgroup

Among the 19 patients with obstructive coronary artery disease, 10 patients (52.6%) had at least one severe occlusive coronary lesion, defined as either a chronic total occlusion (CTO) or a subtotal stenosis. A CTO was present in 8 of 19 patients (42.1%), while 5 of 19 patients (26.3%) had at least one subtotal stenosis. Three patients (15.8%) had both CTO and subtotal stenosis documented in different coronary vessels. At the lesion level, 11 CTOs and 5 subtotal stenoses were recorded. CTOs were most frequently located in the right coronary artery (*n* = 6), followed by the circumflex artery (*n* = 3) and the left anterior descending artery (*n* = 2). Subtotal stenoses were documented in the circumflex artery (*n* = 2), right coronary artery (*n* = 2), and left anterior descending artery (*n* = 1). Severe occlusive lesions in patients with obstructive CAD are summarized in Table 3. Vessel-level distribution of severe occlusive lesions is summarized in Table 4.

### 3.5. Coronary Revascularization

Among the 19 patients with obstructive CAD, 9 (47.4%) underwent percutaneous coronary intervention during the index coronary angiography, 9 (47.4%) underwent coronary artery bypass grafting, and 1 patient (5.3%) was managed with medical therapy without revascularization.

## 4. Discussion

The present study provides descriptive evidence that obstructive coronary artery disease was documented in a minority of patients presenting with a dilated cardiomyopathy phenotype who were referred for first-time diagnostic coronary angiography.

In this Bulgarian single-center cohort, obstructive CAD was present in 19 of 107 patients, corresponding to a prevalence of 17.8%. In contrast, the vast majority of patients had non-obstructive or no significant epicardial coronary stenoses. These findings indicate that obstructive epicardial CAD was not identified in the majority of this selected angiography-referred cohort; however, coronary anatomy alone cannot establish a non-ischemic etiology.

The relatively young age of the present cohort and its derivation from an invasive angiography-referred population should be considered when interpreting the observed prevalence of obstructive CAD. Because the likelihood of coronary atherosclerosis increases with age and cardiovascular risk burden, the 17.8% prevalence observed in this study should not be extrapolated to unselected populations with left ventricular systolic dysfunction or a DCM phenotype. The exceptionally high prevalence of hypertension in our cohort (98.1%) should be interpreted in the context of the selected tertiary care, angiography-referred population and may reflect the cardiovascular risk profile of patients referred for invasive evaluation. This further limits generalizability to unselected DCM populations.

At the same time, the finding that nearly one in five patients had obstructive CAD remains clinically relevant. This supports the continued role of coronary angiography in selected patients presenting with a dilated cardiomyopathy phenotype, particularly when symptoms, cardiovascular risk profile, electrocardiographic abnormalities, regional wall-motion abnormalities, or other imaging findings raise suspicion of ischemic heart disease. In such patients, identification of obstructive CAD may alter etiologic classification, suggest an ischemic or mixed phenotype, and influence subsequent management.

In clinically stable patients in whom an invasive strategy is not immediately required, coronary CT angiography may provide a non-invasive approach for assessment of coronary anatomy, whereas stress imaging may provide complementary functional information regarding inducible ischemia. Invasive coronary angiography remains particularly relevant when the clinical suspicion of significant CAD is high, non-invasive findings are inconclusive, or knowledge of coronary anatomy is expected to influence a potential revascularization strategy [5]. Earlier diagnostic studies showed that ischemic and non-ischemic cardiomyopathy cannot always be reliably distinguished on clinical or echocardiographic grounds alone; tissue characterization by cardiovascular magnetic resonance may provide complementary information when coronary anatomy and the ventricular phenotype are discordant [1,3,8,9].

Cardiovascular magnetic resonance was not performed in any patient in the present cohort and therefore was not available for etiologic classification. In patients with non-obstructive coronary anatomy, CMR could have provided complementary tissue characterization and helped identify alternative non-ischemic myocardial substrates [1,3,8,9]. Importantly, the angiographic presence of obstructive CAD does not by itself establish that left ventricular dysfunction is ischemic in origin [10,11]. In the present study, regional wall-motion abnormalities were not systematically available, and therefore concordance between the distribution of coronary artery disease and the pattern of left ventricular dysfunction could not be assessed. Etiologic classification carries prognostic significance, and prior work has shown that the extent of CAD, together with a history of myocardial infarction or revascularization, improves classification of ischemic cardiomyopathy; isolated single-vessel disease without previous infarction or revascularization may have different etiologic and prognostic implications from multivessel disease [12,13]. Accordingly, the present study reports the prevalence and angiographic extent of obstructive CAD rather than attempting definitive adjudication of ischemic cardiomyopathy.

Serial assessment of left ventricular function may provide additional etiological and prognostic information, particularly because the degree of functional recovery may differ according to the underlying substrate. Follow-up LVEF was not systematically available in the present cohort; therefore, the relationship between coronary anatomy and subsequent left ventricular reverse remodeling could not be assessed. Consistent with the potential prognostic relevance of serial ventricular assessment, Perea-Armijo et al. reported that patients with HFrEF who achieved improvement in LVEF had a lower prevalence of ischemic etiology than those with sustained reduced LVEF (22.2% vs. 42.2%) and who experienced fewer heart failure readmissions and lower mid-term mortality [14].

The angiographic distribution observed in the present cohort also suggests heterogeneity in coronary disease burden. Although single-vessel and two-vessel disease were relatively uncommon, three-vessel disease was present in 12 patients and accounted for 63.2% of all obstructive CAD cases. This indicates that obstructive CAD in this cohort was frequently multivessel rather than limited to single-vessel disease.

In addition, more than half of the patients with obstructive CAD had at least one severe occlusive lesion, defined as a CTO or subtotal stenosis, indicating a substantial burden of severe coronary lesions within the obstructive CAD subgroup.

Identifying an ischemic substrate may also have therapeutic consequences, although the benefit of revascularization depends on coronary anatomy, clinical context, and the mode of revascularization. Contemporary reviews emphasize the biological and clinical heterogeneity of ischemic cardiomyopathy [15]. In STICHES, coronary artery bypass grafting added to medical therapy improved long-term outcomes in selected patients with ischemic cardiomyopathy, whereas REVIVED-BCIS2 did not demonstrate a reduction in all-cause death or heart failure hospitalizations with percutaneous coronary intervention in stable patients with severe ischemic left ventricular dysfunction [16,17].

Accordingly, in patients presenting with a DCM phenotype and obstructive CAD, revascularization decisions should be individualized according to coronary anatomy and disease extent, symptom burden, the likelihood that CAD contributes materially to left ventricular dysfunction, the feasibility of revascularization, comorbidities, and procedural risk. Patients with extensive multivessel disease considered for CABG may represent a substantially different clinical population than stable patients considered for PCI, and the presence of obstructive CAD alone should not be interpreted as an indication for revascularization.

Our findings are broadly consistent with previous angiography-based studies, although important differences in study populations should be considered. N’Guetta et al. included 108 patients with DCM undergoing coronary angiography in Côte d’Ivoire; the median age was 52 years, 75% were male, and hypertension was present in 53.7%. DCM was defined using indexed left ventricular dilatation and an LVEF < 50%, while obstructive CAD was defined as ≥70% stenosis in an epicardial coronary artery or ≥50% in the left main artery; obstructive CAD was identified in 21.3% of patients [6]. Chandra et al. prospectively enrolled 209 consecutive patients in North India with NYHA class II–III dyspnea, global left ventricular hypokinesia, and LVEF ≤ 40% and excluded patients with angina, prior CAD, ischemic ECG findings, or regional wall-motion abnormalities. Their cohort had a mean age of 54.1 years, was 83.7% male, and had substantially higher rates of diabetes and smoking than the present cohort; significant CAD was identified in 21.1% [7]. Thus, although the observed prevalence of obstructive or significant CAD was similar across the three studies, differences in inclusion criteria, cardiovascular risk profiles, and referral strategies limit direct comparison.

The relatively low prevalence of obstructive CAD in this selected cohort indicates that most patients referred for invasive evaluation did not have angiographically significant epicardial coronary disease. The high prevalence of cardiovascular risk factors observed in our cohort is also in line with Bulgarian epidemiological data showing a persistent clustering of major cardiovascular risk factors among urban hypertensive patients [18]. Nevertheless, the identification of obstructive CAD in nearly one in five patients remains clinically important, particularly because it may alter etiologic interpretation and subsequent management.

The non-obstructive group was also heterogeneous: 27 patients had angiographically visible non-obstructive coronary atherosclerosis, whereas 61 had no angiographic stenoses. Beyond the presence or absence of flow-limiting stenosis, coronary atherosclerotic burden may also have prognostic implications in patients with a non-ischemic DCM phenotype. Canu et al. reported that higher coronary atherosclerotic burden may carry prognostic significance in non-ischemic dilated cardiomyopathy, suggesting that coronary disease in these patients may not always be an incidental finding [19]. Histopathologic work by Repetto et al. likewise showed that coronary atherosclerosis may coexist with idiopathic DCM, supporting the concept that coronary disease can represent a comorbid or bystander substrate rather than the sole cause of ventricular dysfunction [20]. Accordingly, angiographically visible non-obstructive coronary atherosclerosis should not be regarded as equivalent to normal coronary arteries and should prompt attention to cardiovascular risk factor optimization and appropriate preventive management, while evaluation for alternative causes of the DCM phenotype continues.

The current work may serve as a useful basis for a larger institutional registry integrating angiographic findings with echocardiographic characteristics, heart failure severity, cardiovascular risk factors, and medical therapy. Such a dataset would allow more precise characterization of the ischemic and non-ischemic spectrum of DCM and would support more robust prognostic analyses.

## 5. Limitations

This study has several limitations. First, its retrospective single-center design introduces the possibility of referral bias, because only patients referred for invasive coronary angiography were included. Therefore, the results should not be interpreted as representing the prevalence of obstructive CAD among all patients with a dilated cardiomyopathy phenotype, but rather among those selected for angiographic evaluation in a tertiary-care clinical setting. Second, the analysis was based on registry-derived categorical angiographic data and procedural documentation rather than on systematic central angiographic adjudication. A formal assessment of inter-observer variability was not performed. Third, the definition of obstructive CAD was based on angiographic stenosis severity and did not include methods for systematic physiological assessment. Finally, additional clinical, echocardiographic, therapeutic, and follow-up variables beyond those summarized in Table 1 were not uniformly available for all patients. Therefore, comparisons between patients with and without obstructive CAD should be regarded as descriptive rather than definitive. The cohort allowed a descriptive estimate of obstructive CAD prevalence, but the 95% confidence interval around the observed prevalence of 17.8% (11.7–26.1%) remains relatively wide. The estimate should therefore be interpreted as a center-specific estimate rather than as a precise population prevalence. The small obstructive CAD subgroup (*n* = 19) also limits the precision of subgroup comparisons and precludes more extensive multivariable analyses.

The long inclusion period and individualized referral strategy may have introduced temporal and selection bias. However, coronary anatomical assessment in the included patients was performed exclusively with invasive coronary angiography rather than coronary CT angiography. Revascularization practice also evolved during the study period, and treatment strategies were not standardized across the entire inclusion period.

Regional wall-motion abnormalities were not systematically available, precluding assessment of the anatomical concordance between coronary lesions and the pattern of left ventricular dysfunction.

Cardiovascular magnetic resonance was not performed in this cohort, precluding tissue characterisation that might have facilitated further etiologic classification, particularly in patients without obstructive CAD.

Follow-up LVEF was not systematically available, precluding the assessment of left ventricular functional recovery or reverse remodeling according to coronary disease status.

## 6. Conclusions

In this retrospective single-center cohort of patients presenting with a dilated cardiomyopathy phenotype and referred for first-time diagnostic coronary angiography, obstructive CAD was identified in 17.8% of patients. Most patients had no obstructive epicardial CAD. However, when obstructive CAD was present, three-vessel disease and severe occlusive lesions were frequent. These findings underscore the clinical role of coronary anatomical assessment in selected patients with a DCM phenotype when an ischemic or mixed etiology is suspected.

## Figures and Tables

**Table 1:** Baseline characteristics of the study cohort according to obstructive coronary artery disease status.

Variable	Overall Cohort (*n* = 107)	Non-Obstructive/No Significant CAD (*n* = 88)	Obstructive CAD (*n* = 19)	*p*-Value
Age, years	55.4 ± 10.9	54.8 ± 10.7	58.3 ± 11.7	0.245
Male sex	97 (90.7%)	78 (88.6%)	19 (100.0%)	0.203
Hypertension	105 (98.1%)	86 (97.7%)	19 (100.0%)	1.000
Diabetes mellitus	22 (20.6%)	14 (15.9%)	8 (42.1%)	0.024
Dyslipidemia	81 (75.7%)	63 (71.6%)	18 (94.7%)	0.039
Active smoking	18 (16.8%)	17 (19.3%)	1 (5.3%)	0.187
Alcohol use	19 (17.8%)	18 (20.5%)	1 (5.3%)	0.185
eGFR, mL/min/1.73 m^2^	82.4 ± 22.5	84.2 ± 21.2	74.1 ± 27.0	0.138
LDL cholesterol, mmol/L	2.69 ± 1.05	2.73 ± 1.08	2.48 ± 0.91	0.299
LVEDD, mm	65 ± 6.4	64.9 ± 6.5	65.5 ± 6.1	0.610
LVEF, %	33.6 ± 8.4	33.9 ± 8.2	32.4 ± 9.2	0.531

Abbreviations: CAD, coronary artery disease; eGFR, estimated glomerular filtration rate; LDL, low-density lipoprotein; LVEDD, left ventricular end-diastolic diameter; LVEF, left ventricular ejection fraction.

**Table 2:** Angiographic distribution of coronary artery disease in the study cohort.

Category	*n*	% of Total Cohort (*n* = 107)	% of Obstructive CAD Subgroup (*n* = 19)
No angiographic stenoses	61	57.0%	-
Non-obstructive coronary atherosclerosis	27	25.2%	-
Single-vessel disease	3	2.8%	15.8%
Two-vessel disease	4	3.7%	21.1%
Three-vessel disease	12	11.2%	63.2%
Obstructive CAD total	19	17.8%	100%

**Table 3:** Severe occlusive lesions in patients with obstructive CAD.

Variable	*n*	% of Obstructive CAD Subgroup (*n* = 19)
Patients with ≥1 severe occlusive lesion	10	52.6
Patients with ≥1 CTO	8	42.1
Patients with ≥1 subtotal stenosis	5	26.3
Patients with both CTO and subtotal stenosis	3	15.8

**Table 4:** Vessel-level distribution of severe occlusive lesions.

Vessel	CTO Lesions, *n*	Subtotal Stenoses, *n*
LAD	2	1
LCx	3	2
RCA	6	2
Total	11	5

## Data Availability

The data presented in this study are available from the corresponding author upon reasonable request. The data are not publicly available due to privacy and ethical restrictions.
